# Constrictive bronchiolitis and paraneoplastic pemphigus caused by unicentric Castleman disease in a young woman: a case report

**DOI:** 10.3389/fmed.2024.1468251

**Published:** 2024-10-15

**Authors:** Ruxuan Chen, Yaqun Teng, Yinbo Xiao, Lu Zhang, Xianlin Han, Weibin Wang, Zhaohui Lu, Xinlun Tian

**Affiliations:** ^1^Department of Pulmonary and Critical Care Medicine, Peking Union Medical College Hospital, Chinese Academy of Medical Sciences and Peking Union Medical College, Beijing, China; ^2^Department of Internal Medicine, Peking Union Medical College Hospital, Chinese Academy of Medical Sciences and Peking Union Medical College, Beijing, China; ^3^Department of Pathology, Peking Union Medical College Hospital, Chinese Academy of Medical Sciences and Peking Union Medical College, Beijing, China; ^4^Department of Hematology, Peking Union Medical College Hospital, Chinese Academy of Medical Sciences and Peking Union Medical College, Beijing, China; ^5^Department of General Surgery, Peking Union Medical College Hospital, Chinese Academy of Medical Sciences and Peking Union Medical College, Beijing, China

**Keywords:** Castleman disease, paraneoplastic pemphigus, paraneoplastic autoimmune multiorgan syndrome, constrictive bronchiolitis, lung transplantation, case report

## Abstract

**Introduction:**

Constrictive bronchiolitis is a rare and severe condition characterized by progressive and irreversible obstruction of small airways. Constrictive bronchiolitis could be part of paraneoplastic autoimmune multiorgan syndrome secondary to Castleman disease.

**Case description:**

A 20-year-old female presented with progressive exertional dyspnea and severe obstructive ventilatory dysfunction. She also experienced recurrent and painful oral mucosal erosions. Upon investigation for underlying conditions, contrast-enhanced CT imaging revealed a pelvic mass exhibiting marked enhancement and hypertrophied vessels. A diagnosis of Castleman disease was confirmed via ultrasound-guided percutaneous biopsy of the pelvic tumor. Autoantibodies indicative of paraneoplastic pemphigus were detected using indirect immunofluorescence on rat bladder tissue. Complete surgical resection of the pelvic mass was undertaken with the collaborative efforts of a multidisciplinary team. Despite resolution of mucocutaneous lesions, symptoms of constrictive bronchiolitis persisted after the surgery. Subsequently, the patient underwent lung transplantation and demonstrated significant improvement in lung function.

**Conclusion:**

Timely diagnosis and comprehensive multidisciplinary management of this rare and life-threatening syndrome are crucial for enhancing patient outcomes.

## 1 Introduction

Castleman disease (CD) is a rare and heterogenous group of nonclonal lymphoproliferative disorder, which is further classified as “unicentric” when it occurs in a single lymph node station (1). Although most unicentric Castleman disease (UCD) patients have favorable prognosis following resection of the enlarged lymph node, a small subset of patients with paraneoplastic pemphigus (PNP) and constrictive bronchiolitis (CB) face poor prognosis and high mortality (1, 2). Constrictive bronchiolitis, also known as obliterative bronchiolitis or bronchiolitis obliterans, is a rare and severe condition characterized by progressive and irreversible obstruction of small airways (3, 4). CB is often misdiagnosed as other obstructive airway disorders, such as chronic obstructive pulmonary disease (COPD) and asthma, while progressive CB can lead to respiratory failure and death. Timely and accurate diagnosis and management of CB remain significant clinical challenges.

In this case report, we describe the critical condition of a young woman with cough, progressive exertional dyspnea and oral mucosal erosions. She was ultimately diagnosed with UCD complicated by PNP and CB. We detail the diagnostic process, including differential diagnosis, and discuss the clinical management led by a multidisciplinary team, which ultimately resulted in a favorable outcome for the patient.

## 2 Case description

**Figure 1:**
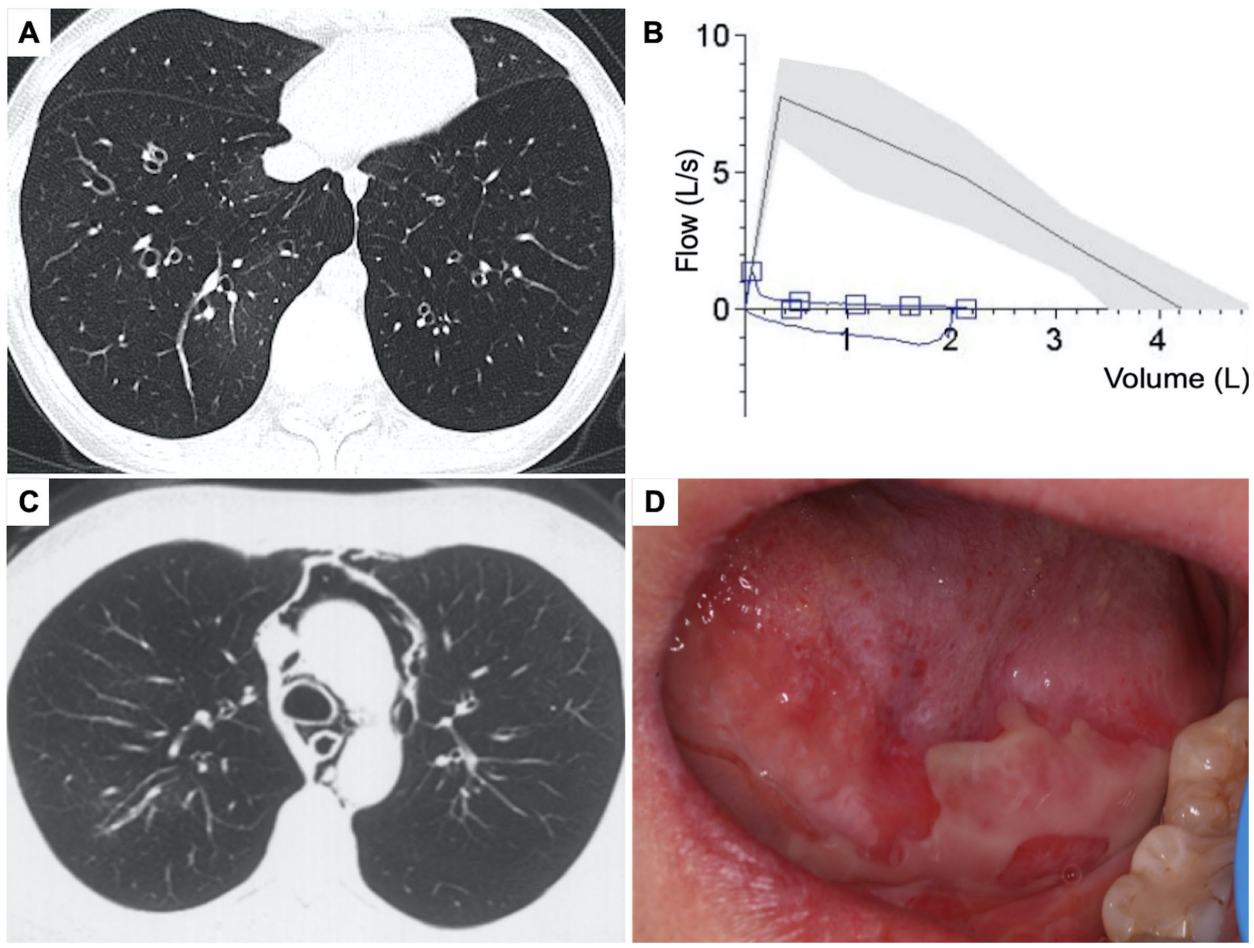
Clinical presentation of the patient. (A) Chest high resolution CT, axial view showed diffuse bronchiectasis with patchy ground-glass opacity and pulmonary hyperinflation. (B) Pulmonary function test showed severe obstructive dysfunction. (C) Chest CT showed spontaneous pneumomediastinum. (D) Physical sign of oral mucosal erosions on the tongue.

A previously healthy 20-year-old woman presented to our center with a 10-month history of a persistent cough and progressive exertional dyspnea. High-resolution computed tomography (HRCT) of the chest revealed diffuse bronchiectasis, patchy ground-glass opacities, and pulmonary hyperinflation (Figure 1A). Pulmonary function tests (PFT) demonstrated severe obstructive ventilatory dysfunction (ratio of forced expiratory volume in 1 s [FEV_1_] to forced vital capacity [FVC] was 21%; FEV_1_ was 0.45 L, 12% of the predicted value) and no response to bronchodilator (Figure 1B), along with a markedly increased residual volume (4.89 L, 312%). With an elevated serum total immunoglobulin (Ig) E level of 1,639 U/mL, she has been diagnosed with severe allergic asthma. However, despite repeated treatments with intravenous corticosteroids, inhaled bronchodilators and antibiotics, her symptoms worsened, and her physical tolerance significantly deteriorated. One month prior to admission, she experienced a spontaneous pneumomediastinum (Figure 1C) and subcutaneous emphysema. Additionally, she had been suffering from persistent and painful oral mucosal erosions for the past 18 months, which had severely affected her appetite and led to a weight loss of 15 kg. She had no rashes, abdominal pain, fever, or night sweats. She denied smoking or exposure to toxic gasses, and her family history was unremarkable.

On admission, her vitals were normal, and pulse oxygen saturation of 98% on room air. Multiple oral mucosal erosions were observed on the tongue (Figure 1D), inner side of both the upper and lower lips, and buccal mucosa. No conjunctival or anogenital lesions were noted. She had no skin lesions. On pulmonary auscultation, wheezes were identified at the end of expiration bilaterally. Cardiac examination showed regular heart rhythm without any murmurs. Her abdomen was tender and painless, and no definitive mass was found on palpation. She had no peripheral enlarged lymph nodes or edema.

Laboratory investigations, including a complete blood count, metabolic profile, and inflammatory biomarkers, were within normal limits. The serum aspergillus-specific IgE tests were negative, which did not support the diagnosis of allergic bronchopulmonary aspergillosis. A mildly positive serum antinuclear antibody (ANA) was noted at a titer of 1:80, but specific autoantibodies associated with connective tissue diseases were absent. Screening for pemphigus-related autoantibodies, including BP180, BP 230, Dsg1, and Dsg3, yielded negative results. No serum monoclonal proteins were detected.

**Figure 2:**
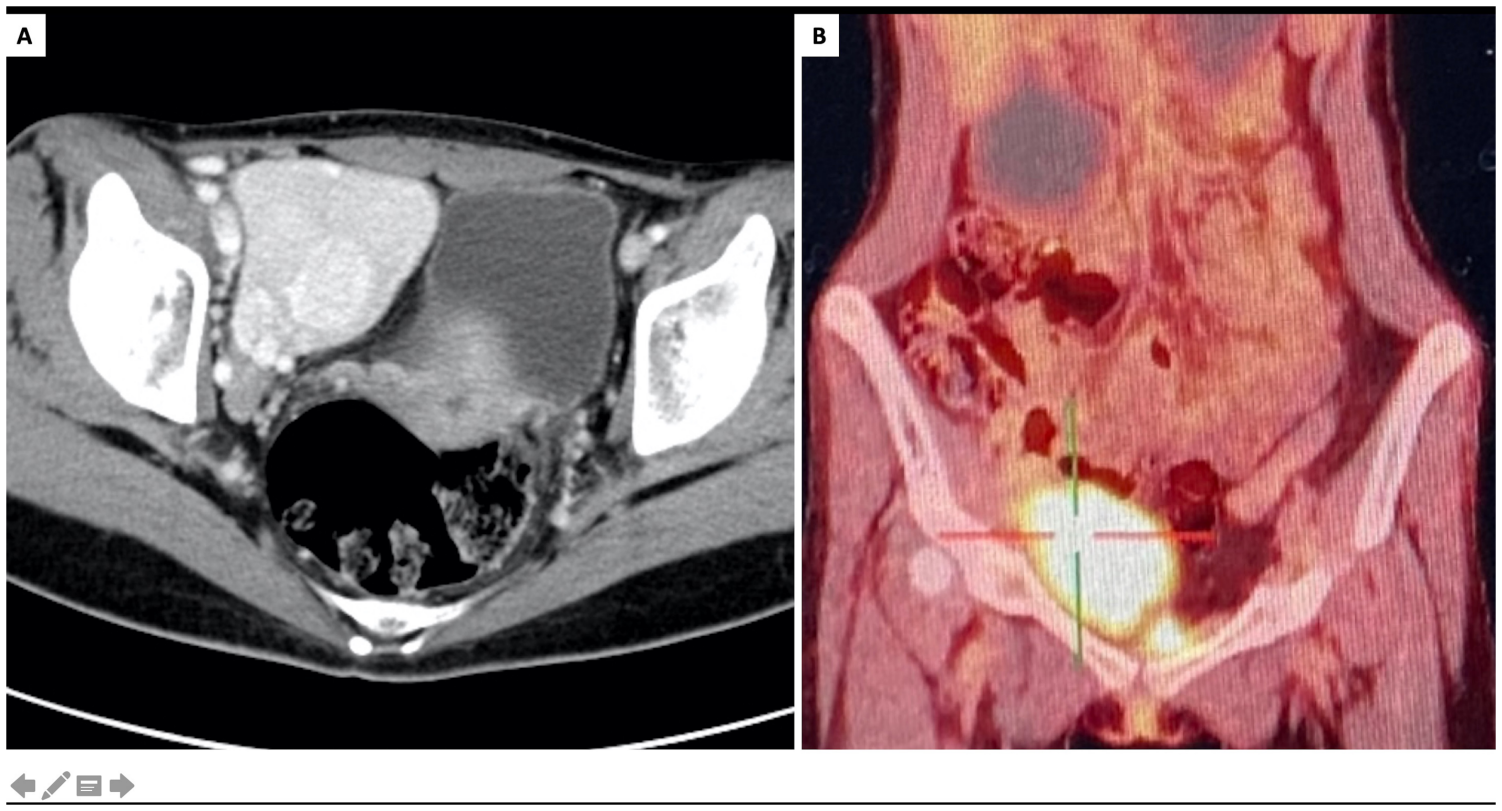
Characterization of the pelvic mass. (A) Contrast-enhanced CT, axial view showed a pelvic tumor at right iliac fossa with marked enhancement and hypertrophied vessels. (B) PET/CT scan, coronary view showed increased metabolic activity of the pelvic tumor.

Considering clinical diagnosis of constrictive bronchiolitis, a contrast-enhanced CT scan was conducted to screen underlying tumors. A pelvic mass exhibiting pronounced enhancement and hypertrophied vessels was observed (Figure 2A). Positron emission tomography (PET) scan revealed increased metabolic activity in the pelvic mass, along with multiple hypermetabolic lymph nodes adjacent to the bilateral iliac vessels (Figure 2B). Further, the oral mucosal erosions were highly indicative of paraneoplastic pemphigus, which was later confirmed by positive tests of indirect immunofluorescence (1:320, intercellular IgG deposition on the rat bladder epithelial cells).

Since the pelvic tumor was considered as the culprit of PNP and CB, its pathological examination was vital for diagnosis. Since the pelvic mass has a rich blood supply, the risk of bleeding from biopsy is high. Additionally, the proximity of the pelvic mass to the iliac vessels, nerves, and right ureter raises the risk of tissue damage during biopsy or surgical removal. Furthermore, the patient’s severe obstructive pulmonary dysfunction presented a significant risk associated with general anesthesia during surgery.

**Figure 3:**
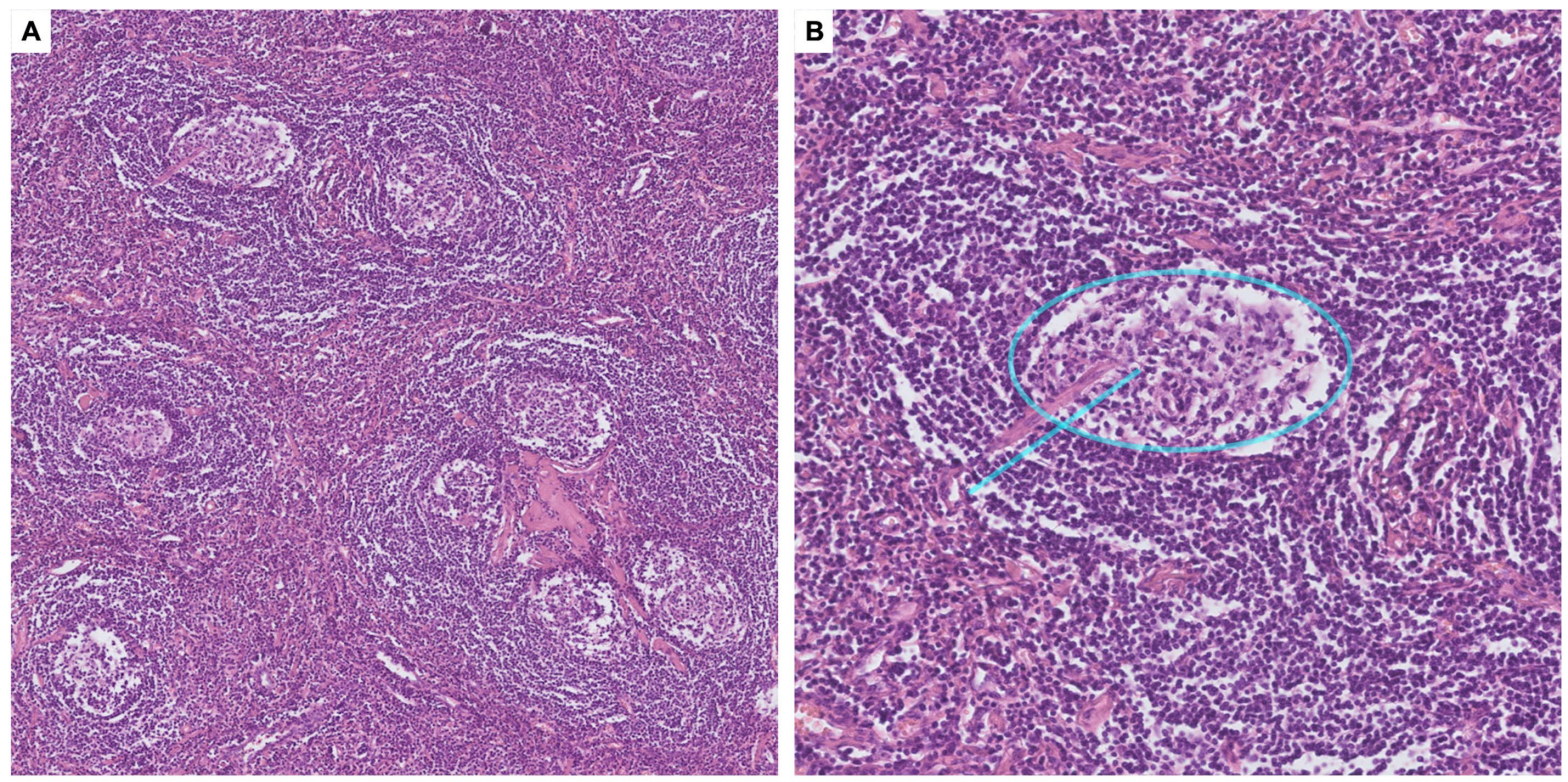
Pathological findings of the pelvic tumor. (A) Microscopy with ×10 magnification, H&E staining. Pathology of the pelvic tumor showed germinal centers atrophy, mantle zones expansion in an “onion-skin pattern,” and increased vascularity with hyalinization. (B) Microscopy with ×40 magnification, H&E staining. Interfollicular vascular proliferation with conspicuous hyalinization penetrating the germinal centers, presenting a “lollipop” appearance (blue line).

Following thorough discussions by a multidisciplinary team (MDT), an ultrasound-guided percutaneous biopsy of the pelvic tumor was performed, which indicated a pathological diagnosis of CD. Given that complete surgical resection is the key treatment strategy for UCD, the multidisciplinary team decided to proceed with surgical resection of the tumor, alongside necessary perioperative preparations. For treatments of CB, inhaled corticosteroid and bronchodilators, oral prednisone (15 mg per day) and azithromycin were administrated. Intravenous immunoglobulin was used in the perioperative period to reduce the risk of exacerbation of CB. An interventional embolization was performed before the surgery to reduce the risk of bleeding by blocking the main blood supply to the pelvic mass. A Double-J stent was inserted into the right ureter to minimize the risk of ureteral injury during surgery. Finally, the pelvic tumor resection surgery was successfully performed under neuraxial anesthesia. The surgical specimen consisted of several fused lymph nodes with increased numbers of lymphoid follicles, and the pathological examination confirmed diagnosis of hyaline vascular CD (Figure 3). No evidence of monoclonal lymphoproliferative disease was detected.

**Figure 4:**
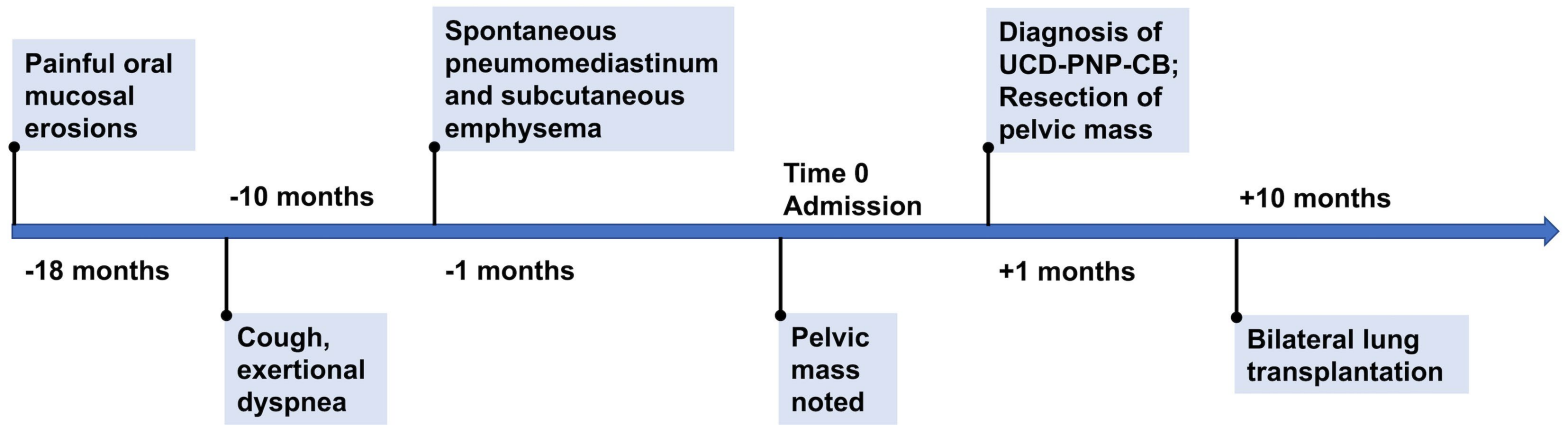
Timeline of the case. UCD, unicentric Castleman disease. PNP, paraneoplastic pemphigus. CB, constrictive bronchiolitis.

After surgery, the patient reported diminished oral mucosal erosions, moderately alleviated respiratory symptoms, and improved physical tolerance. Nevertheless, episodes of dyspnea persisted, triggered by intermittent upper respiratory tract infections, and no significant improvement in pulmonary function was observed over an eight-month follow-up. At last, the patient underwent bilateral lung transplantation in January 2024, and exhibited favorable postoperative recovery. At the six-month follow-up after lung transplantation in July 2024, the patient was generally in good condition, and had essentially normal pulmonary function. The timeline of this case was shown in Figure 4.

## 3 Discussion

Initially presented with common symptoms, the patient was ultimately diagnosed with a rare condition: constrictive bronchiolitis caused by Castleman disease. The crucial step in this case was the differential diagnosis of her primary symptoms of chronic cough and dyspnea. A chest CT scan ruled out overt lung parenchyma damage, and PFT revealed severe and nonreversible obstruction of the small airways. Given that the patient is a young woman without smoking history or other exposures, common airway diseases such as COPD were unlikely due to the absence of risk factors. Severe asthma was also ruled out due to the persistent and nonreversible nature of the airway obstruction and the lack of responsiveness to large dose steroid treatment. Bronchiolitis encompasses a wide spectrum of inflammatory and fibrosing processes affecting the bronchioles, and this was supported by signs of bronchiolar wall thickening, bronchiolectasis, and expiratory air trapping observed on HRCT scans (4). CB is a histological subtype of bronchiolitis characterized by subepithelial acellular fibrosis within the bronchiole walls, leading to concentric narrowing or obliteration of the small airways (3, 4). The patient’s clinical history of dry cough and progressive dyspnea, along with radiographic features and PFT results, were consistent with CB (5). Notably, the presence of pneumomediastinum and interstitial emphysema further supported the diagnosis of CB (4).

CB could be secondary to a variety of etiologies: post allogeneic hematopoietic stem-cell transplantation (HSCT) or lung transplantation, exposures of inhalational toxins, post infections, and autoimmune disorders (3, 4). Paraneoplastic pemphigus (PNP), also known as paraneoplastic autoimmune multiorgan syndrome (PAMS), is a severe mucocutaneous blistering disease triggered by paraneoplastic autoimmunity involving both humoral and cell-mediated immune mechanisms (6). Patients of PNP typically present with severe oral mucositis with or without polymorphous skin lesions, while CB is the most frequent extracutaneous complication of PNP (~30%) that contributes to high mortality (6, 7). The final diagnosis of PNP is made by detection of autoantibodies to plakin family proteins and by the positive reactivity with rat bladder by indirect immunofluorescence (8). In this patient, the recurrent oral mucosal erosion presented a clinical clue of PNP, which prompted the screening of underlying tumor. Although available tests for pemphigus-related autoantibodies (including BP180, BP 230, Dsg1, and Dsg3) were negative, the diagnosis of PNP was finally confirmed by the presence of pelvic tumor and positive tests of indirect immunofluorescence on rat bladder. The pathogenic IgG autoantibodies in PNP are polyclonal and the reported autoantibody profile is heterogeneous. Indirect immunofluorescence with mouse or rat bladder as a substrate has a higher sensitivity (86%) and specificity (98.9%) for the diagnosis of PNP (9).

Lymphoproliferative disorders constitute the majority of the underlying neoplasms in patients with PNP, including non-Hodgkin lymphoma (52.8%), chronic lymphocytic leukemia (22.9%), and Castleman disease (18.6%) (7, 8). Importantly, Castleman disease is a common cause of PNP in patients complicated with CB, especially in children and adolescents, and Asian populations (1, 8, 10). Originally termed “giant lymph node hyperplasia,” UCD is commonly manifested as an enlarging mass with radiological signs of calcification, marked enhancement and hypertrophied vessels (11). The incidence of UCD has been estimated to be between 0.6 and 16 cases per million person-years (2). PNP and CB are rare manifestations of UCD, which also associate with poor prognosis and high mortality risks (7). In the largest multicenter study of CD in China led by our center, 903 patients of UCD were involved, 53 patients (5.9%) had documented PNP, and 35 patients (3.9%) had CB (12). Histopathology subtypes of CD include hyaline vascular (HV), plasma cell (PC), and mixed-type of both HV and PC (1, 2). In HV subtype, the histopathology features are follicular hyperplasia, expansions of mantle zones forming concentric rings of small lymphoid cells (the so-called “onion skin pattern”), and atrophic germinal centers, which could be penetrated by pathological blood vessels (the “lollipop lesions”). In PC subtype, the key pathological feature is the infiltration of sheets of plasma cells in the interfollicular zone. In this patient, diagnosis of hyaline vascular CD is finally confirmed by the pathological examination of the affected lymph node.

Complete surgical excision is the most effective treatment for UCD, and usually halts or reverses the associated PNP (1). However, CB is hardly reversable, with its progression being the most common cause of death in patients with UCD (1, 7). Besides, surgical excision of pelvic UCD in patients with PNP and CB is associated with substantial perioperative risks. Therefore, a significant clinical challenge is determining whether surgical treatment is necessary for UCD patients with concomitant PNP and CB, and whether it will provide actual prognostic benefits for the patient. As illustrated in this case, resecting the UCD allowed for control of PNP and provided the necessary time and conditions to prepare for a lung transplant, ultimately improving her long-term prognosis (13). The MDT comprising experts in Pulmonary and Critical Care Medicine, Hematology, Pathology, General Surgery, Anesthesiology, Interventional Radiology, Urology, and Intensive Care Unit provided crucial guidance throughout the patient’s diagnosis and treatment. We therefore recommend that the optimized surgical plan and perioperative management should be tailored for patients with UCD complicated by PNP and CB by the MDT. It is worth noting that CB could develop after surgical resection of primary tumor or worsen after the surgery (14). Possible explanation was proposed that the tumor is usually rich in blood vessels, and crash of tumor during the operation results in the massive release of pathogenic antibodies. Administration of intravenous immunoglobulin during the preoperative period could help to neutralize the circulatory antibodies and avoid CB flare following surgery (15).

## 4 Conclusion

CB is a critical and life-threatening condition characterized by the progressive obstruction of the small airways. Early diagnosis of CB is crucial and can be achieved through the identification of patients exhibiting progressive dyspnea, obstructive airflow impairment in PFT, and signs of air trapping and bronchiectasis in HRCT. Mucocutaneous lesions in patients of CB are important clues for PNP and should prompt screening of the underlying tumor. Castleman disease is an important cause of PNP and CB, particularly among young individuals and those in Asian populations. Complete surgical resection of the enlarged lymph node is the most important treatment of UCD patients with PNP. Although CB may not be reversible following UCD resection, lung transplantation remains a vital option for restoring pulmonary function and improving prognosis. A multidisciplinary team should be established to direct the optimized treatment of patients with UCD complicated by PNP and CB.

## Data Availability

The original contributions presented in the study are included in the article/supplementary material, further inquiries can be directed to the corresponding author.

## References

[1] van Rhee F Oksenhendler E Srkalovic G Voorhees P Lim M Dispenzieri A. International evidence-based consensus diagnostic and treatment guidelines for unicentric Castleman disease. Blood Adv. (2020) 4:6039–50. doi: 10.1182/bloodadvances.2020003334, PMID: 33284946 PMC7724917

[2] Carbone A Borok M Damania B Gloghini A Polizzotto MN Jayanthan RK. Castleman disease. Nat Rev Dis Primers. (2021) 7:84. doi: 10.1038/s41572-021-00317-7, PMID: 34824298 PMC9584164

[3] Barker AF Bergeron A Rom WN Hertz MI. Obliterative bronchiolitis. N Engl J Med. (2014) 370:1820–8. doi: 10.1056/NEJMra1204664, PMID: 24806161

[4] Poletti V Ravaglia C Dubini A Kronborg-White S. Cazzato S. Piciucchi S. “Bronchiolitis”. In: TOF Wagner Humbert M Wijsenbeek M Kreuter M Hebestreit H Rare diseases of the respiratory system (ERS monograph). Sheffield, European Respiratory Society (2023). p. 85–102.

[5] Devakonda A Raoof S Sung A Travis WD Naidich D. Bronchiolar disorders: a clinical-radiological diagnostic algorithm. Chest. (2010) 137:938–51. doi: 10.1378/chest.09-0800, PMID: 20371529

[6] Anderson HJ Huang S Lee JB. Paraneoplastic pemphigus/paraneoplastic autoimmune multiorgan syndrome: part I. Clinical overview and pathophysiology. J Am Acad Dermatol. (2024) 91:1–10. doi: 10.1016/j.jaad.2023.08.020, PMID: 37597771

[7] Ouedraogo E Gottlieb J de Masson A Lepelletier C Jachiet M Salle de Chou C. Risk factors for death and survival in paraneoplastic pemphigus associated with hematologic malignancies in adults. J Am Acad Dermatol. (2019) 80:1544–9. doi: 10.1016/j.jaad.2018.03.043, PMID: 30981429

[8] Kim JH Kim SC. Paraneoplastic pemphigus: paraneoplastic autoimmune disease of the skin and mucosa. Front Immunol. (2019) 10:1259. doi: 10.3389/fimmu.2019.01259, PMID: 31214197 PMC6558011

[9] Maruta CW Miyamoto D Aoki V Carvalho RGR Cunha BM Santi CG. Paraneoplastic pemphigus: a clinical, laboratorial, and therapeutic overview. An Bras Dermatol. (2019) 94:388–98. doi: 10.1590/abd1806-4841.20199165, PMID: 31644609 PMC7007015

[10] Mimouni D Anhalt GJ Lazarova Z Aho S Kazerounian S Kouba DJ. Paraneoplastic pemphigus in children and adolescents. Br J Dermatol. (2002) 147:725–32. doi: 10.1046/j.1365-2133.2002.04992.x, PMID: 12366419

[11] Zhao S Wan Y Huang Z Song B Yu J. Imaging and clinical features of Castleman disease. Cancer Imaging. (2019) 19:53. doi: 10.1186/s40644-019-0238-0, PMID: 31345268 PMC6659281

[12] Zhang L Dong YJ Peng HL Li H Zhang MZ Wang HH. A national, multicenter, retrospective study of Castleman disease in China implementing CDCN criteria. Lancet Reg Health West Pac. (2023) 34:100720. doi: 10.1016/j.lanwpc.2023.100720, PMID: 37283978 PMC10240357

[13] Yue B Huang J Jing L Yu H Wei D Zhang J. Bilateral lung transplantation for Castleman disease with end-stage bronchiolitis obliterans. Clin Transpl. (2022) 36:e14496. doi: 10.1111/ctr.14496, PMID: 34590355

[14] Zhang J Qiao QL Chen XX Liu P Qiu JX Zhao H. Improved outcomes after complete resection of underlying tumors for patients with paraneoplastic pemphigus: a single-center experience of 22 cases. J Cancer Res Clin Oncol. (2011) 137:229–34. doi: 10.1007/s00432-010-0874-z, PMID: 20390428 PMC11827967

[15] Tang JQ Chen HK Wang X Wang MY Xiong Y Wang H. Retrospective analysis of 45 cases of localized retroperitoneal Castleman disease from a single center. Hepatobiliary Surg Nutr. (2020) 9:304–11. doi: 10.21037/hbsn.2019.05.05, PMID: 32509816 PMC7262626

